# Colocynth Oil, a Substitute for Croton Oil in Neuralgia, Sciatica, &c.

**Published:** 1841-01

**Authors:** M. G. Janelli


*Colocynth Oil, a Substitute for Croton Oil in Neuralgia, Sciatica, &c.* By M. G. Janelli.—Dr. Janelli mentions that he has found the oil of colocynth a valuable and cheap succedaneum for croton oil, as an external application in neuralgic affections, but especially in sciatica. He relates in detail three cases of its efficacy in sciatica, and three in cases of rheumatism. After frictions with the oil, the patient generally fell asleep, so much were their sufferings alleviated, and in a few days were so far recovered as to be enabled to return to their usual avocations.—*Observatore Medico. Ib.*

